# Emerging Trends in the Epidemiology of West Nile and Usutu Virus Infections in Southern Europe

**DOI:** 10.3389/fvets.2019.00437

**Published:** 2019-12-06

**Authors:** Tatjana Vilibic-Cavlek, Vladimir Savic, Tamas Petrovic, Ivan Toplak, Ljubo Barbic, Dusan Petric, Irena Tabain, Ivana Hrnjakovic-Cvjetkovic, Maja Bogdanic, Ana Klobucar, Anna Mrzljak, Vladimir Stevanovic, Petra Dinjar-Kujundzic, Luka Radmanic, Federica Monaco, Eddy Listes, Giovanni Savini

**Affiliations:** ^1^Department of Virology, Croatian Institute of Public Health, Zagreb, Croatia; ^2^School of Medicine, University of Zagreb, Zagreb, Croatia; ^3^Poultry Center, Croatian Veterinary Institute, Zagreb, Croatia; ^4^Department for Virology, Scientific Veterinary Institute, Novi Sad, Serbia; ^5^Veterinary Faculty, University of Ljubljana, Ljubljana, Slovenia; ^6^Department of Microbiology and Infectious Diseases With Clinic, Faculty of Veterinary Medicine, University of Zagreb, Zagreb, Croatia; ^7^Laboratory for Medical and Veterinary Entomology, Faculty of Agriculture, University of Novi Sad, Novi Sad, Serbia; ^8^Center for Microbiology, Institute of Public Health Vojvodina, Novi Sad, Serbia; ^9^Medical Faculty, University of Novi Sad, Novi Sad, Serbia; ^10^Division of Disinfection, Disinfestation and Pest Control, Andrija Stampar Teaching Institute of Public Health, Zagreb, Croatia; ^11^Department of Medicine, Merkur University Hospital, Zagreb, Croatia; ^12^OIE Reference Center for West Nile Disease, Istituto Zooprofilattico Sperimentale “G. Caporale”, Teramo, Italy; ^13^Laboratory for Diagnostics, Croatian Veterinary Institute, Regional Institute Split, Split, Croatia

**Keywords:** West Nile virus, Usutu virus, epidemiology, “One Health”, Southern Europe

## Abstract

The epidemiology of West Nile (WNV) and Usutu virus (USUV) has changed dramatically over the past two decades. Since 1999, there have been regular reports of WNV outbreaks and the virus has expanded its area of circulation in many Southern European countries. After emerging in Italy in 1996, USUV has spread to other countries causing mortality in several bird species. In 2009, USUV seroconversion in horses was reported in Italy. Co-circulation of both viruses was detected in humans, horses and birds. The main vector of WNV and USUV in Europe is *Culex pipiens*, however, both viruses were found in native *Culex* mosquito species (*Cx. modestus, Cx. perexiguus*). Experimental competence to transmit the WNV was also proven for native and invasive mosquitoes of *Aedes* and *Culex* genera (*Ae. albopictus, Ae. detritus, Cx. torrentium*). Recently, *Ae. albopictus* and *Ae. japonicus* naturally-infected with USUV were reported. While neuroinvasive human WNV infections are well-documented, USUV infections are sporadically detected. However, there is increasing evidence of a role of USUV in human disease. Seroepidemiological studies showed that USUV circulation is more common than WNV in some endemic regions. Recent data showed that WNV strains detected in humans, horses, birds, and mosquitoes mainly belong to lineage 2. In addition to European USUV lineages, some reports indicate the presence of African USUV lineages as well. The trends in WNV/USUV range and vector expansion are likely to continue in future years. This mini-review provides an update on the epidemiology of WNV and USUV infections in Southern Europe within a multidisciplinary “One Health” context.

## Introduction

West Nile virus (WNV) and Usutu virus (USUV) are mosquito-borne flaviviruses characterized by similar clinical manifestations and overlapping geographic distribution, host and vector species. The epidemiology of WNV and USUV has changed dramatically over the past two decades. Since 1996, an increasing number of WNV outbreaks in humans and horses were detected, and the area of its circulation expanded in many Southern European countries (1). After USUV emergence in Austria in 2001, it subsequently spread to neighboring countries causing mortality in several wild bird species, mainly Eurasian blackbirds (2). However, a retrospective analysis of archived bird tissue samples from Italy (Tuscany region) in 1996 identified USUV, indicating a much earlier introduction of this virus into Europe (3).

Since the early 2000s, WNV circulation has been continuously monitored in some European countries with varying number of human and horse cases. USUV infections are reported in birds, while human clinical cases are rarely detected (4–6). However, there is increasing evidence of a role of USUV in human disease. Seroepidemiological studies showed that USUV circulation in humans is more common than WNV in some endemic regions where both viruses circulate (7).

Until the introduction of WNV lineage 2 in 2004 (8), WNV lineage 1 was identified as the cause of human outbreaks in Europe. In the following years, lineage 2 dispersed to the eastern part of Austria and to Southern European countries (9–11). Recent data showed that strains detected in humans, horses, birds, and mosquitoes mainly belong to WNV lineage 2 (6, 12, 13). Although the majority of USUV strains belong to European USUV lineages, some reports indicate the presence of African USUV lineages as well (5).

In 2018, WNV infections in Europe increased dramatically compared to previous transmission seasons. A total of 2,083 human cases and 285 outbreaks among equids were reported with the largest number detected in Italy, Serbia, and Greece (14). USUV was detected in asymptomatic blood donors, birds, and mosquitoes in Italy (15, 16) and in three patients with neuroinvasive disease in Croatia (6).

The Middle East represents an important transit zone for bird migration between Africa and Eurasia and provides valuable information on circulation of WNV/USUV in these regions (17–19). In Israel, serologic evidence and acute WNV infections have been reported in humans, horses, birds, and mosquitoes (20, 21). While strains sequenced from humans mainly belonged to the WNV lineage 1 (22), mosquito surveillance revealed a high genetic diversity of WNV (23). USUV strains detected in mosquitoes belonged to a putative novel lineage Europe 5 (24). WNV/USUV infections were also reported in other countries bordering the Mediterranean Sea (France, Turkey, Cyprus) (5, 25–40). This mini-review provides an update on the epidemiology/molecular epidemiology of WNV and USUV infections in Southern Europe and neighboring countries within a multidisciplinary “One Health” context.

**Figure 1:**
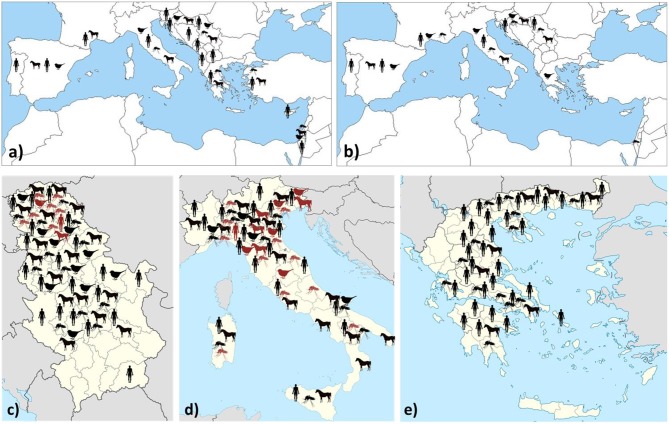
Geographic distribution of WNV **(a)** and USUV **(b)** infections/seropositivity in southern Europe and neighboring countries. Regional distribution of WNV (black) and USUV (red) infections (clinical cases/IgM detection/seroconversion/RNA detection) in countries with a high prevalence: Serbia **(c)**, Italy **(d)**, and Greece **(e)**.

Data on WNV/USUV infections are presented in Table 1 and Figure 1.

**Table 1:**
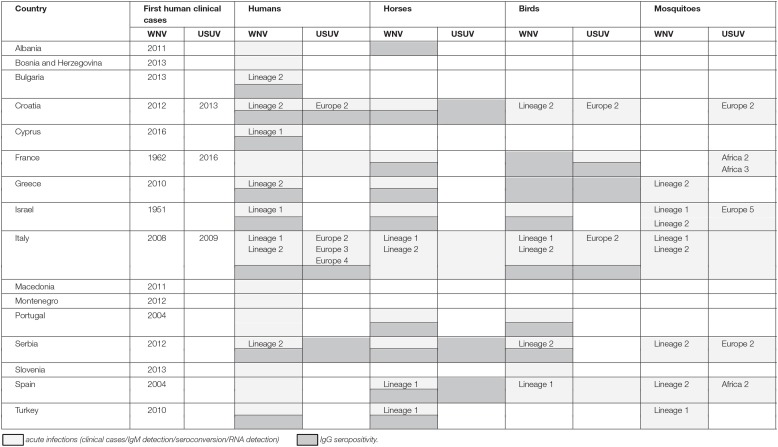
Summary of West Nile and Usutu virus infections in Southern Europe.

*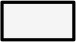 acute infections (clinical cases/IgM detection/seroconversion/RNA detection) 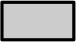 IgG seropositivity*.

## Albania

Data on WNV infections in Albania are scarce. To date, only two human cases were reported in 2011 (14). A serological study conducted among horses from 12 districts in Albania showed WNV seropositivity of 22.2%, with significant regional differences (7.7–66.7%). The highest seroprevalence rates were reported in districts near the Mediterranean Sea, whereas low or negative seroprevalence was detected in districts located further inland (41). Data on USUV infection are not available.

## Bosnia and Herzegovina

There is only one report, in 2013, of two cases of WNV neuroinvasive disease (WNND) in Bosnia and Herzegovina (detected in the Tuzla and Kladanj region). Three out of nine patients screened for WNV met clinical criteria for WNND, of which two had high serum IgM titres. RT-PCR for WNV RNA was negative. Since the neutralization test was not performed, cases were classified according to the European Union case definition as probable WNV (42). So far, there are no data on USUV infections in Bosnia and Herzegovina.

## Bulgaria

In 2015, the first confirmed human case with fatal outcome reported in Bulgaria was caused by a Central/Southern-European lineage 2 WNV (43). Human cases were continuously detected in the following seasons (12). A nationwide study conducted among residents of all 28 districts in Bulgaria revealed a 1.5% WNV seroprevalence with the highest seropositivity (up to 10%) detected in districts near the Danube River (44). USUV infections were not documented in Bulgaria.

## Croatia

The first outbreak of human WNND in Croatia was detected in 2012 in eastern counties, thereafter outbreaks (2013, 2017) and sporadic cases (2014–2016) were continuously notified in continental Croatia (4, 45). The largest outbreak was recorded in 2018 with 54 cases of WNND and 7 cases of WNV fever in 10 of the 21 counties (6). During the 2013 WNV outbreak, the first three cases of neuroinvasive USUV disease were detected in Zagreb and surrounding areas (4, 46). Three additional USUV cases were confirmed in the 2018 outbreak, of which one was fatal (6). A serological study conducted from 2010 to 2011 showed WNV antibodies in 3.43% horses and 0.11% cattle with the highest seropositivity in eastern counties bordering Hungary and Serbia (47). Seropositivity and acute asymptomatic WNV infections in horses were detected continuously in subsequent years. Seroprevalence varied greatly by year and region (0–26%) with the highest seropositivity in counties with documented human cases (4, 48). Although passive monitoring of WNV in birds was established in Croatia in 2012, WNV infections were not detected until the summer of 2018, when WNV infection was confirmed serologically in one buzzard presenting with neurological symptoms, and WNV RNA was detected in two dead goshawks from Northwest Croatia. Two USUV seropositive horses were documented in 2011 in Northwest Croatia (49). In 2018, USUV RNA was detected in one dead blackbird in Zagreb County (6). To date, none of the mosquito pools tested were WNV RNA positive. However, USUV-positive mosquito pools were found in 2016 (*Aedes albopictus)*, 2017 (*Culex pipiens*), and 2018 (*Cx. pipiens*) in Northwestern counties (6, 50). Sequenced strains from humans, wild birds, and mosquitoes (2018) confirmed circulation of WNV lineage 2 and USUV Europe 2 lineage (6).

## Greece

No WNV clinical cases in humans or horses had been reported in Greece prior to the large WNV outbreak in 2010 when 262 human cases with extremely high fatality rates (17%) were reported mostly in Central Macedonia (51). The outbreaks continued in 2011 and 2012 in areas that had not been affected before (52, 53). Thereafter, outbreaks occurred in humans every year except 2015 and 2016 (14, 54). The Greek WNV strains detected during 2010–2018 clustered within the Central/Southern European subclade of lineage 2 (55). In 2018, a novel genetic variant was detected that belonged to the Eastern European subclade of lineage 2 (56). A nationwide WNV seroprevalence study (2013) showed seropositivity of 1.5% in the Greek population (57), whilst WNV antibodies were detected in 4% of horse serum samples (2001–2008) (58). In 2010, WNV infection was reported in 17 horses with neurological symptoms (59). Furthermore, a high WNV seropositivity (18.6%) was reported in cattle (60). In Central Macedonia, pigeon seroprevalence was 54 and 31% at the end of the 2010 and 2011 epidemic seasons, respectively, while one serum was positive for USUV neutralizing antibodies (61). A small-scale entomological study performed in Central Macedonia, at the epicenter of the 2010 WNV outbreak found two positive *Cx. pipiens* pools. Following this large epidemic, an active mosquito surveillance system was implemented in Greece for a 3-year period (2011–2013). Positive *Cx. pipiens* pools were detected in different areas of the country and preceded the diagnosed human cases (62). WNV lineage 2 was detected in *Cx. pipiens* mosquito pools during the 2017 outbreak (63).

## Italy

A multi-species national surveillance plan was implemented by the Italian government in 2002, following the first WNV outbreak (64). In the following years, the program has been adapted according to the WNV new epidemiological scenarios and a National Integrated Plan Monitoring USUV and WNV is in place since 2016. Human WNV outbreaks were continuously notified since 2008 (14). WNV lineage 1 was responsible for reported WNND cases in 2010. In 2011–2012, lineage 1 and 2 co-circulated with a higher proportion of lineage 1 and from 2013 to 2016 lineage 2 was most prevalent (64–66). From 2017, only lineage 2 has been detected. Since 2008, WNV infections were continuously detected also in horses and birds (65). WNV lineage 2 was confirmed in wild birds (2014) (11, 67) and horses with fatal WNV neuroinvasive infection (68). The presence of USUV in humans was serologically confirmed for the first time in four blood donors in 2009 (69). A survey conducted retrospectively on cerebrospinal fluid (CSF) and serum samples in Modena (2008–2011) found USUV RNA in 1.1% CSF samples, while WNV RNA was not detected. USUV antibody levels were significantly higher (6.57%) compared to that of WNV (2.96%) indicating that USUV infection is not a sporadic event in humans in Italy (70). A very high USUV seroprevalence was found in forestry workers (18.1%) (71). Neuroinvasive USUV infection was detected for the first time in Italy in two immunocompromised patients in 2009 (72, 73). During 2017–2018, USUV Europe 2, 3, and 4 lineages were confirmed in blood donations in the Lazio region (15). USUV neutralizing antibodies were detected in horses with a higher seropositivity in 2008 (89.2%) compared to 2009 (7.8%). In the same study, USUV RNA was found in blackbirds and magpies (Emilia Romagna and Veneto). Additionally, USUV neutralizing antibodies were detected in rock pigeon, blackbird and, magpie (74). *Culex pipiens* is the mosquito species most involved in the WNV and USUV circulation in Italy, although other species would also support the spread of both viruses during winter months (75). An entomologic investigation conducted in 2013 showed WNV RNA in 1.9% and USUV in 2.6% mosquito pools. Each virus was detected mainly in *Cx. pipiens* pools; however some pools tested positive for both viruses. The majority of the WNV strains detected in mosquitoes belonged to WNV lineage 2, while WNV lineage 1 which predominantly circulated in 2008–2012, was still detected at low levels until 2016 (64, 76). Sequence analysis of the first known isolate of USUV to cause human encephalitis indicated European USUV lineage (77). The whole genome sequences of USUV strains isolated from mosquitoes and wild birds in Northern Italy (2010–2014) showed Europe 2 and Europe 4 lineages, respectively (78).

## Portugal

In the summer of 2004, two human cases of WNV infection were reported in Portugal in tourists (Ria Formosa, Algarve) (79). Shortly after this report, a WNV monitoring program was established. The presence of WNV was assessed by serological surveys in horses (2004–2010), wild birds, and birds from zoological parks. Detection of WNV antibodies in horses and birds in all the years covered by the study as well as the presence of WNV IgM antibodies in horses with neurological signs supported the evidence of WNV circulation in Portugal (80). However, no human clinical cases were reported in the country from 2005 to 2010. In 2010, another WNV human case was identified (81). Following a 5-year period of apparent absence, WNV re-emerged in southern Portugal in 2015 when WNND was diagnosed in a man from the Algarve region (82). There are no data on USUV infections in Portugal.

## Serbia

The serological surveys (2005–2010) revealed the presence of WNV IgG antibodies in 3.99% human serum samples in Vojvodina Province (northern Serbia), with varying yearly rates (1.97–6.04%) (83). The first outbreak of human WNND was detected in 2012 (Belgrade and Vojvodina) with 69 reported and 41 clinically and laboratory confirmed cases (14, 84). Each year thereafter, seasonal outbreaks were observed. In 2013, 303 cases were reported from 18/25 districts in Serbia; 202 WNND cases were confirmed with lethality of 11.6%. From 41 to 76 WNND cases were documented annually between 2014 and 2017. The largest epidemic so far was recorded in 2018 with 415 WNND cases detected in 13/25 districts (14). A serological study conducted in 2009–2010 showed for the first time WNV neutralizing antibodies in 12% of horses from Vojvodina (85). Seropositivity and asymptomatic WNV infections in horses were detected continuously in the subsequent years; 28.6% seroprevalence from 2010 to 2011 and 49.23% in 2012 in Vojvodina (86, 87). In 2014, seroconversion was detected in 2.57% horses from the whole country. In addition, in 2015, 2017, and 2018, acute WNV infections were detected in 0.53, 0.41, and 1.47% of horses tested, respectively (88–90). WNV antibodies were detected in 7.6% wild birds sampled from 2011 to 2012 in Vojvodina, of which 133 birds were found dead. Virus presence was confirmed in 9.87% of tissue samples and in the blood sample of one bird (91). From 2014 onwards, WNV was detected in wild birds, with the highest percentage of positive samples being observed in 2018 when WNV was detected in 11.61% of tissues and 6.56% of pharyngeal swabs (88–90). All WNV isolates belonged to WNV lineage 2 (88, 91). Additionally, WNV antibodies were identified in 15.4% farm pigs, 17.6% wild boars, and 18.7% roe deer sampled from 2011 to 2012 (Vojvodina) (92). WNV was detected in mosquitoes for the first time in 2010 in the city of Novi Sad. At that time, 3/841 mosquito pools were positive by WNV specific RT-PCR. The pools originated from 66 localities in 29 settlements in Vojvodina (60). More than 9% of the mosquito pools examined during 2012–2013 were positive for WNV (93). Thereafter, country-wide surveys detected WNV in 2.31, 2.09, and 2.51% of *Cx. pipiens* during 2014, 2015, and 2017, respectively, whereas 12.21% of *Cx. pipiens* tested positive in 2018. Other naturally-infected mosquito species were *Ae. vexans* and *Culiseta annulata* (83). All isolates from mosquitoes (2010–2018) were shown to belong to WNV lineage 2 (83, 88–92, 94–96). USUV neutralizing antibodies were detected in 1/349 horses (2009-2010), and 4/318 wild boars (2011–2012) in Vojvodina (85, 92). Additionally, USUV RNA was confirmed in 0.4% of *Cx. pipiens* pools from Vojvodina in 2014 (74). In 2015, USUV was detected in 0.93% of *Cx. pipiens* pools while USUV antibodies were detected in 5% of human serum samples from South Bačka District (Vojvodina) (97). Moreover, in 2017, USUV RNA was detected in 2.75% of *Cx. pipiens* mosquitoes collected in Vojvodina. Two isolates were typed as USUV Europe 2 lineage (98). Thus, far, USUV has not yet been detected in wild birds in Serbia.

## Slovenia

Only four human cases of WNV infections were reported in Slovenia so far. The first case was confirmed in 2013 in a patient with meningitis. Thereafter, in 2018, three cases of WNND were confirmed by detection of IgM antibodies in the CSF samples (99). In 2009, 481/1912 (25.2%) clinically healthy horses were positive for WNV IgG antibodies (100). From 2003 until 2009, serum samples from 34 species of songbirds were tested with WNV seroprevalence of 4.7% (101). Furthermore, from 2010 to 2012, wild bird carcasses were collected through passive monitoring of wild bird mortality and tested for WNV RNA, however, there was no evidence of wild bird mortality due to WNV (102). As well, there was no evidence of USUV circulation in Slovenia.

## Spain

The first human clinical case of WNV infection was diagnosed in 2004 in a patient with aseptic meningitis visiting Southwestern Spain (103). Autochthonous cases were subsequently notified in 2010, 2016, and 2018 transmission seasons (14, 104). WNV cases in horses caused by WNV lineage 1 were continuously reported since 2010 (105). A seroprevalence study conducted in equine populations of Mallorca Island (2011–2012) showed seropositivity rates to WNV and USUV of 6.4 and 1.2%, respectively (106). Another study conducted among horses from central Spain (2011–2013) found WNV seroprevalence of 1.35% (107). WNV lineage 1 was isolated from diseased and dead golden eagles in 2007 (108). In 2011, a survey conducted in waterfowl used as decoys in Andalusia showed that the frequency of seropositive decoys ranged 1.5–3.1% for WNV and 4.4–5.9% for USUV (109). Additionally, WNV and USUV antibodies were found in 23 and 10% of hunted wild red-legged partridges and pheasants from Cádiz (2011–2012) (110). In 2012, USUV was confirmed in two song thrushes (111). WNV and USUV antibodies were also detected in wild birds (2013) (112) as well as pigeons and zoo birds from Cordoba (2013–2014) (113). Moreover, USUV Africa 2 lineage in 2006 (Northeastern Spain) and 2009 (Southern Spain) as well as WNV lineage 1 in 2008 (Southern Spain) were detected in mosquitoes (114, 115).

## Conclusions and Future Perspectives

An integrated “One Health” surveillance of mosquitoes and birds in several countries has proven to be useful for early detection of WNV/USUV circulation and identification of enzootic areas (48, 61, 116). Virus detection in mosquitoes and birds preceded human and horse cases (48, 62). In addition, a high seroprevalence in sentinel animals was detected in areas with documented human cases (4, 22, 48, 61).

There are still many challenges in the epidemiology of WNV/USUV. In addition to birds, which are well-known reservoirs, WNV and USUV antibodies were found in different animal species. WNV and USUV neutralizing antibodies were documented in red deer in Spain (117). WNV seropositive dogs were detected in Italy, Spain, and Corsica Island (118), while USUV antibodies were found in hunting dogs in Italy (119). Moreover, WNV and USUV antibodies were found in gray squirrels in Italy, broadening the host range for these viruses (120). WNV neutralizing antibodies were also found in two wild rodents (*Apodemus flavicollis*) captured in forested areas of Italy (121). However, the short-term and low-level viremia makes it unlikely that these animal species play a role in the WNV transmission cycle. *Culex pipiens* mosquitoes appear to be a major vector for WNV and USUV transmission in Europe, but *Cx. modestus* and *Cx. perexiguus* play an important role in marshlands of some southern countries (115). However, detection of WNV and USUV in different field-collected native *(Ae. vexans*) and invasive (*Ae. albopictus, Ae. japonicus*) mosquito species indicates their possible role in promoting the overwintering of these viruses (75, 83, 122–125). Additionally, a recently published study identified *Cx. torrentium* as a highly competent vector for WNV in Central and Northern Europe (126).

While several licensed veterinary WNV vaccines are currently available, there is no WNV or USUV vaccine for humans (127, 128). Since data from Europe indicate that both WNV and USUV appear to be expanding their geographical ranges and the trends indicate that this spread is likely to continue in future years, the development of an effective vaccine is urgently needed to protect at-risk populations from neurological complications.

## Author Contributions

TV-C and TP made contributions to conception and design of the study, involved in data collection, and drafting the manuscript. VSa, LB, ITa, IH-C, MB, AK, AM, VSt, PD-K, LR, FM, and EL were involved in data collection and drafting the manuscript. ITo, DP, and GS revised the manuscript critically. All authors read and approved the final manuscript.

### Conflict of Interest

The authors declare that the research was conducted in the absence of any commercial or financial relationships that could be construed as a potential conflict of interest.
